# Body mass index and Hodgkin's lymphoma: UK population-based cohort study of 5.8 million individuals

**DOI:** 10.1038/s41416-019-0401-1

**Published:** 2019-02-27

**Authors:** Helen Strongman, Adam Brown, Liam Smeeth, Krishnan Bhaskaran

**Affiliations:** London School of Hygiene and Tropical Medicine, London, UK

**Keywords:** Hodgkin lymphoma, Risk factors, Epidemiology, Obesity

## Abstract

Previous epidemiological studies describe a positive association between body mass index (BMI) and Hodgkin’s lymphoma, mainly in obese vs. normal weight individuals. We examined the shape of this relationship in individuals aged 16 years or older, using primary care data from the United Kingdom’s Clinical Practice Research Datalink. Cox models were fitted with linear, non-linear (spline) and categorical BMI. Models were adjusted for potential confounders and effect modification was investigated. Five point eight two million patients were included, 927 of whom developed Hodgkin’s lymphoma during 41.6 million years of follow-up. Each 5 kg/m^2^ increase in BMI was associated with a 10% increase in Hodgkin’s lymphoma (95% confidence intervals: 2–19). Analysis of non-linearity suggested a J-shaped association with incidence increasing with BMI above 24.2 kg/m^2^. Seven point four per cent of adult Hodgkin’s lymphoma cases were estimated to be attributable to excess weight. Our findings suggest a pattern of increasing risk beyond the World Health Organisation healthy weight category in the general population.

## Introduction

Obesity can modify immune function and thus may increase risk of Hodgkin's lymphoma (HL).^1^ Evidence suggests a positive association between body mass index (BMI) and HL, but there have been few studies, and limited investigation into the shape of the relationship. Our group has previously characterised associations between BMI and 22 common cancers.^2^ We have extended this work to test the association between BMI and incident HL using the same methodology and population-based primary care database (Clinical Practice Research Datalink (CPRD) GOLD) to the previous study.^2^

## Methods

The CPRD GOLD database includes information collected during primary care consultations in the United Kingdom (UK) National Health Service to inform future clinical care. This includes BMI measurements collected following registration with the current or former practice, or during medical visits, either opportunistically or to inform specific medical decisions.^3^ Variables indicating the start of patient registration at the CPRD practice and start of continuous recording of data at the practice are used to define the start of research quality follow-up. We included individuals in CPRD aged 16 years or older with any BMI measurement recorded, eligible research quality follow-up time and no previous cancer diagnosis. BMI was calculated directly from height and weight records (weight/height^2^). If BMI was recorded at the start of research quality follow-up, study follow-up started 12 months after the first BMI measurement to avoid reverse causality. If not, follow-up started 12 months after the start of registration and the most recent previous BMI (if available) was used. This was time updated when the first research standard BMI was recorded. Outcomes were ascertained using clinical codes indicating malignant HL diagnosis. Follow-up ended at lymphoma diagnosis or end of patient or practice data collection.

We assessed linear and non-linear (restricted cubic spline) associations of BMI with HL. Cox proportional hazards models with attained age as the underlying time-scale were adjusted for covariates at first BMI measurement: age (restricted cubic spline), smoking status, alcohol use, previous diabetes diagnosis, index of multiple deprivation, calendar period; stratified by sex. Patients with missing data for smoking or alcohol status were removed. Effect modification by sex, age (time updated) and smoking status was investigated using interaction terms and restriction to non-smokers. The proportional hazards assumption was investigated using Schoenfeld residuals. The population attributable fraction was calculated by fitting a three-category BMI variable (BMI < 18.5, 18.5–25,  ≥25 kg/m^2^), and combining estimated hazard ratios (HRs) for overweight and obesity with national prevalence (Health Survey for England 2010). The results of Cox proportional hazards models with BMI measured in World Health Organisation (WHO) categories are also reported to allow for comparison with previous publications. The study was approved by the London School of Hygiene and Tropical Medicine Ethics Committee and the pre-specified scientific protocol was approved by the Independent Scientific Advisory Committee for MHRA Database Research (protocol 12_090A3).

## Results

The initial study population included 6.33 million individuals aged 16 years or older in CPRD, with eligible follow-up time and no cancer diagnosis before cohort entry. These individuals had a mean first BMI of 25.6 kg/m^2^ (SD 5.2); 3.45 million (54.5%) subjects were female. People in higher BMI categories were older (median age 42.7 years among overweight/obese individuals vs. 37.4 among healthy weight individuals), less likely to be current smokers (overweight/obese: 32.1% vs. healthy weight: 36.2%), and more likely to have had a diabetes diagnosis (overweight/obese: 5.0%; healthy weight: 1.6%). Mean duration of follow-up was 8.9 years (SD 6.5) from the first BMI record. There were some missing data for smoking status (0.8%) and alcohol use (7.9%).

Of 5.82 million patients with complete data, 927 developed HL during 41.6 million years of follow-up. Each 5 kg/m^2^ linear increase in BMI was associated with the same estimated 10% increase in HL in crude and adjusted models (hazard ratio (HR) 1.10, 99% confidence interval (CI): 1.02–1.19) providing strong evidence of a positive association between BMI and incident HL.

**Fig. 1:**
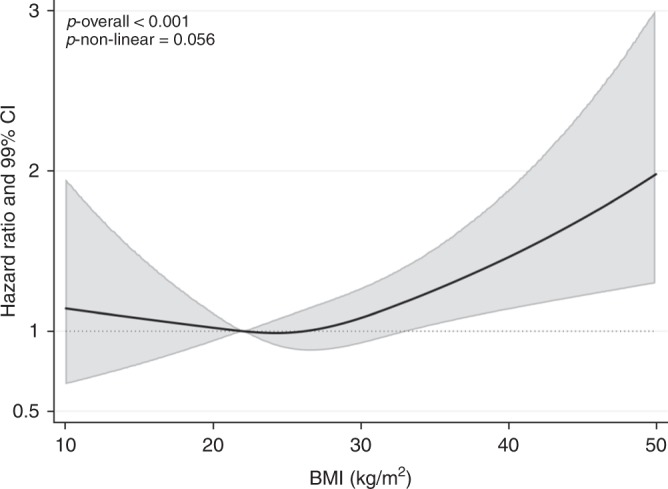
Association between body mass index (BMI) and Hodgkin's lymphoma (HL), allowing for non-linear effects, with 99% confidence intervals (CIs). The reference BMI (with HR fixed as 1.0) was 22 kg/m^2^. The model included a 3-knot restricted cubic spline for BMI (knots placed at equal percentiles of BMI), adjusted for age (3-knot restricted cubic spline), calendar year, diabetes status, alcohol use, smoking, geographical area-based deprivation (index of multiple deprivation), and stratified by sex. HR = hazard ratio

Figure 1 shows the estimated shape of the BMI–HL association using flexible spline models. The association appears to be J-shaped with HL risk increasing above 24.3 kg/m^2^. There was strong evidence of association (*p* < 0.001) and a suggestion of non-linearity (*p* = 0.06). We found no evidence of effect modification by sex, age or smoking status, and no evidence against the proportional hazards assumption (*p* = 0.37). Crude and adjusted models with BMI represented in WHO categories provide weak evidence of an association with obesity (adjusted HR 1.19, 99% CI: 0.94–1.50) and no evidence of an association with overweight (adjusted HR 0.99, 99% CI: 0.81–1.21). Under the assumption of causality, we estimated that 7.4% of HL cases were attributed to overweight and obesity.

## Discussion

We found strong evidence of a positive association between BMI and incident HL, with an estimated 10% increase in HL risk per 5 kg/m^2^ increase in BMI (95% CI: 2–19); however, analysis allowing for non-linearity was suggestive of a J-shaped relationship, with positive associations only at BMIs above 24.2 kg/m^2^. Seven point four per cent of adult HL cases were estimated to be attributable to excess weight.

The direction of effect and evidence of non-linearity is consistent with previous studies. However, the effect size is considerably smaller. The recent UK Million Women study indicated a 64% increased risk per 10 kg/m^2^ increase (95% CI: 21–221)^4^ which is equivalent to a 28% increased risk per 5 kg/m^2^ increase (95% CI: 10–49). An earlier meta-analysis of five studies^5^ found strong evidence of a higher risk in obese vs. normal weight individuals (relative risk (RR) 1.41, 95% CI: 1.14–1.75), but no evidence of a higher risk in the overweight category (RR 0.97, 95% CI: 0.85–1.12).

CPRD GOLD is a large data source that has been shown to have a high level of validity generally,^3^ and specifically for cancers^6^ and recently recorded BMI measurements.^7^ Analysing non-linear effects using a spline model allowed a more nuanced picture to emerge than using BMI categories alone and was suggestive of a gradual increase in risk of HL from 24.3 kg/m^2^ rather than the absence of effect in the overweight category (25 to  ≤30 kg/m^2^).

The study also has important limitations. Our study population was restricted to individuals who had BMI, smoking and alcohol status measured in their primary care practice. This would introduce selection bias if the association of BMI, HL and confounders varied between patients with measured and unmeasured BMI, smoking and alcohol status. Previous research and multiple sensitivity analyses in our multi-cancer analysis left us confident that this did not cause major bias.^2,7^ There is also the potential for residual confounding due to unmeasured confounders. These include amount smoked, physical exercise, ethnicity and potential confounders that are specific to HL, for example, Epstein–Barr virus.^8^ Published analyses to date have used BMI data collected for research purposes,^4,9–11^ as part of a mandatory medical examination for a national insurance scheme,^12^ or during a compulsory national screening programme.^13^ Cancer outcomes have primarily been ascertained using cancer registry data. These studies have a similar potential for selection bias or residual confounding, although there are differences in the purpose and therefore completeness of BMI data collection and the availability of data to measure potential confounders.

Inflammation is central to the pathology of HL and associated with adiposity. The association between excess weight and HL may therefore be regulated by the immunomodulatory effect of adipose tissue.^1^ To test this theory, future epidemiological studies should use more precise measures of whole and local body fatness.^1^ Studies could also investigate the impact of duration of obesity and weight change on HL incidence. However, repeated BMI measures and measures of adiposity other than BMI are not yet consistently available in large, population-based databases. As population coverage and completeness of data recording improves, it will also be possible to investigate associations between adiposity and measures of tumour aggressiveness or HL mortality by incorporating linked cancer registration and death data.

In conclusion, our analysis strengthens evidence of a pattern of increased risk of HL with increasing BMI. This supports calls for strategies to prevent excess weight gain beyond the WHO healthy weight category in the general population. Large prospective cohort studies with improved measures of body fat would help elucidate the mechanisms through which adiposity affects HL risk.

## Data Availability

The data that support the findings of this study are available from CPRD, but restrictions apply to the availability of these data, which were used under license for the current study, and so are not publicly available.

## References

[1] Matos A (2016). Mechanisms underlying the association between obesity and Hodgkin lymphoma. Tumor Biol..

[2] Bhaskaran K (2014). Body-mass index and risk of 22 specific cancers: a population-based cohort study of 5.24 million UK adults. Lancet.

[3] Herrett E (2015). Data Resource Profile: Clinical Practice Research Datalink (CPRD). Int J. Epidemiol..

[4] Murphy F (2013). Body size in relation to incidence of subtypes of haematological malignancy in the prospective Million Women Study. Br. J. Cancer.

[5] Larsson SC, Wolk A (2011). Body mass index and risk of non-Hodgkin’s and Hodgkin’s lymphoma: a meta-analysis of prospective studies. Eur. J. Cancer.

[6] Boggon R (2013). Cancer recording and mortality in the General Practice Research Database and linked cancer registries. Pharmacoepidemiol. Drug Saf..

[7] Bhaskaran K, Forbes HJ, Douglas I, Leon DA, Smeeth L (2013). Representativeness and optimal use of body mass index (BMI) in the UK Clinical Practice Research Datalink (CPRD). BMJ Open.

[8] Küppers R (2009). The biology of Hodgkin’s lymphoma. Nat. Rev. Cancer.

[9] Lim U (2007). Alcohol, smoking, and body size in relation to incident Hodgkin’s and non-Hodgkin’s lymphoma risk. Am. J. Epidemiol..

[10] Söderberg KC (2009). Overweight, obesity and risk of haematological malignancies: a cohort study of Swedish and Finnish twins. Eur. J. Cancer.

[11] Yang TO (2016). Body size in early life and risk of lymphoid malignancies and histological subtypes in adulthood. Int. J. Cancer.

[12] Oh SW, Yoon YS, Shin SA (2005). Effects of excess weight on cancer incidences depending on cancer sites and histologic findings among men: Korea National Health Insurance Corporation Study. J. Clin. Oncol..

[13] Engeland A, Tretli S, Hansen S, Bjørge T (2007). Height and body mass index and risk of lymphohematopoietic malignancies in two million Norwegian men and women. Am. J. Epidemiol..

